# Niche Breadth and Olfactory Context Shape Informed Passive Dispersal

**DOI:** 10.1111/ele.70373

**Published:** 2026-03-31

**Authors:** Kamila Zalewska, Anna Skoracka, Dries Bonte, Ewa Puchalska, Mariusz Lewandowski, Lechosław Kuczyński

**Affiliations:** ^1^ Population Ecology Lab, Institute of Environmental Biology, Faculty of Biology Adam Mickiewicz University Poznań Poland; ^2^ Center for Advanced Technology Adam Mickiewicz University Poznań Poland; ^3^ Department of Biology Ghent University Gent Belgium; ^4^ Department of Plant Protection Warsaw University of Life Sciences Warsaw Poland

**Keywords:** experimental evolution, habitat choice, host specialisation, kairomones, niche breadth, olfactory cues, prospecting, wheat curl mite

## Abstract

Animals must decode environmental information to make adaptive decisions. In passive dispersers, for whom only departure timing is under control, the mechanisms of take‐off remain poorly understood. Using experimental evolution, we tested how niche breadth modulates passive dispersal in phytophagous mites by exposing specialist and generalist lineages to host‐derived kairomones. Dispersal was highly context‐dependent. Generalists exhibited higher baseline dispersal and increased take‐off when detecting familiar target cues; however, they were inhibited by complex mixtures of unfamiliar cues (low signal‐to‐noise ratio). Conversely, specialists primarily tracked current host quality, departing significantly more frequently from unfamiliar plants, yet showing little modulation by target cue identity. These results demonstrate that divergent host specialisation in homogeneous versus heterogeneous environments fundamentally alters the way in which organisms integrate information from current and future habitats to drive dispersal. Niche breadth dictates the baseline propensity for informed departure, while olfactory context provides the final trigger for passive take‐off.

## Graphical abstract

Experimental evolution reveals that niche breadth modulates informed passive dispersal in phytophagous mites through divergent strategies. While generalists optimise take‐off based on target cue familiarity and signal‐to‐noise ratios, specialists primarily track current host quality to trigger departure. Niche breadth thus dictates baseline dispersal propensity, while olfactory context provides the final trigger for passive take‐off.

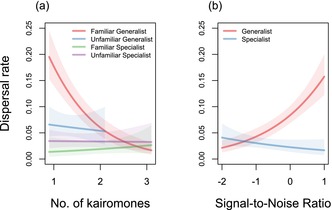

## 1 Introduction

Receiving, decoding and using information correctly is essential for animals to make adaptive decisions. One of the most consequential of these decisions is choosing a suitable habitat. In an ever‐changing environment, animals must continuously assess the quality of their habitat and decide whether to stay or disperse. These choices affect their future interactions with the environmental factors, such as food, shelter, potential mates, competitors and predators. Actively moving species can prospect potential settlement locations and thus evaluate alternative habitats in relation to the current one. Using personal and social information enables them to make informed decisions about departure, movement and settlement (Clobert et al. 2009; Delgado et al. 2014; McPeek et al. 2024; Ponchon and Travis 2022; Thierry et al. 2024; Usinowicz and O'Connor 2023).

While many species actively decide when to depart, some may rely on biotic or abiotic vectors, such as water or air currents, to travel to and colonise new locations. Spiders, for example, engage in passive ballooning, yet they still have to adopt a body position that enables them to be carried by the wind (Cho et al. 2018; Montes and Gleiser 2025). From an individual perspective, passive dispersal is highly risky because transfer phases impose costs such as limited resources and depleted energy when traversing unsuitable habitats. However, these costs may be offset by producing large numbers of dispersing propagules or stages, which allows for bet hedging in uncertain environments. In a heterogeneous landscape with sparsely distributed habitats, the risk of failing to reach a viable site is high and theory predicts that dispersal will be selected against (Bonte et al. 2012; Fronhofer et al. 2024; Henriques‐Silva et al. 2015). Conversely, limited gene flow may increase kin competition or inbreeding, both of which are known to favour dispersal. Importantly, dispersal is rarely a fixed strategy, but rather depends on local environmental factors such as competition. Consequently, dispersal strategies may depend on density (both interspecific and intraspecific), resource availability or body condition, even when the costs are substantial (Bonte et al. 2010, 2012; Gros et al. 2006; Poethke and Hovestadt 2002). To mitigate these risks and optimise the fitness outcomes of dispersal, the evolution of using information would be expected, provided that the benefits of receiving and processing environmental cues outweigh the associated metabolic and sensory costs and that the information itself is reliable (Mortier et al. 2019). Because passively dispersing organisms cannot prospect future habitats, any relevant cues must be perceived and integrated during the departure phase (Bocedi et al. 2012; Delgado et al. 2014; Kiedrowicz et al. 2017).

Overall, dispersal is favoured when the expected fitness benefits of leaving one's place of birth outweigh the costs of staying there. Individuals must therefore weigh the relative advantages of staying versus moving. Individual heterogeneity in these expectations generates variation in dispersal strategies (Bonte et al. 2014; Hamilton and May 1977). As population size increases and resources become limited or the habitat deteriorates, the motivation to disperse is predicted to rise (Banks et al. 2024; Bonte et al. 2012; Laska et al. 2019).

Crucially, fitness expectations depend on the degree to which an organism's phenotype matches its environment, that is, its level of local adaptation. Individuals that are highly adapted to a specific habitat are ecological specialists. Sparse distribution of specialised resources theoretically is expected to result in joint evolution of specialisation and philopatry (Ravigné et al. 2024). Under this view, specialists should exhibit low dispersal to avoid the high risk of landing in unsuitable habitats, whereas generalists, with their broader ecological niches, should disperse more readily to exploit favourable settlement opportunities. However, empirical support is mixed: some studies have confirmed this expectation (e.g., Freedman et al. 2020; Kneitel 2018; Stevens et al. 2012, 2013), while others have reported the opposite or no clear association (e.g., Alzate et al. 2017; Dapporto and Dennis 2013; Van Zandt and Mopper 1998). These inconsistencies likely reflect the frequent neglect of the spatial and temporal dynamics of the conditions to which species have adapted (Ravigné et al. 2024).

For passive dispersers, it is not yet clear whether the degree of specialisation affects emigration decisions, or whether specialists and generalists differ in their ability to perceive and integrate information from both current and potential future habitats. Under optimal conditions, both specialists and generalists could correctly interpret cues from a familiar environment when making dispersal decisions. However, generalists are more likely to land in suitable habitats than specialists, so they may invest less in acquiring information, thereby avoiding the morphological, physiological and temporal costs associated with signal detection and processing. In contrast, specialists are expected to disperse less frequently than generalists and to respond primarily to cues matching their specialised habitat (Bonte et al. 2012). In summary, to determine whether departures in passively dispersing organisms are informed, it is essential to assess how organisms respond to the strength of the signal they receive and to evaluate these responses in the context of both the current and future environments. Without accounting for both, it is impossible to infer whether the information is truly decodable, selective or behaviourally relevant. Furthermore, considering the degree of ecological specialisation provides additional insight into the interplay between specialisation and dispersal and the extent to which information is used.

Our aim is to address these questions and to elucidate the interaction between ecological specialisation and environmental information in modulating informed dispersal decisions. To this end, we employed an experimental approach involving passively dispersing herbivorous arthropods—specifically, the eriophyoid wheat curl mite (*Aceria tosichella* Keifer 1969). We investigated whether dispersal is influenced by:

- Niche breadth, by comparing replicated host‐specialist and flexible host‐use generalist mite lineages obtained through multigenerational experimental evolution.
- Current habitat context, whether the plants on which the mites reside are familiar or unfamiliar based on the mites' evolutionary history.
- Target habitat information, whether olfactory cues emitted by plants are familiar or unfamiliar. We characterised this information using two dimensions:

- Signal Complexity (information quantity, i.e., the number of cues),
- Signal‐to‐Noise ratio (information quality, i.e., the ratio of familiar to unfamiliar cues).

Based on the evolutionary history of mite lineages, we tested four predictions:

1. Because specialists minimise the risk of landing on unsuitable hosts, they will exhibit lower baseline dispersal rates than generalists.
2. Specialists will show higher sensitivity to target cues than generalists, reflecting the greater investment in information processing required by their narrow niche.
3. Dispersal will be highest when mites are in an unfamiliar current environment and encounter familiar target cues.
4. A high information load (high signal complexity) will inhibit dispersal due to sensory processing limitations. In contrast, a high signal‐to‐noise ratio will facilitate an informed departure, particularly for generalists, by providing reliable information about the availability of potential target hosts.

Overall, our results demonstrate that emigration decisions are selective and depend not only on environmental information but are strongly modulated by the organism's degree of host specialisation.

## 2 Material and Methods

### 2.1 The Study System

We used an obligate phytophagous mite *Aceria tosichella* (the wheat curl mite, hereafter WCM), an economically important pest of agricultural crops. The WCM represents a cryptic species complex consisting of several divergent genotypes. Our experiment focused on the MT‐1 genotype, identified through DNA barcoding (Skoracka, Lopes, et al. 2018). The WCM MT‐1 is one of the most serious wheat pests worldwide, causing significant losses through direct feeding and by transmitting plant viruses, which increases its invasive potential and colonisation success. Its short development time (6–7 days at 27°C), rapid multiplication capacity, high dispersal ability and thermal tolerance make it a suitable organism for laboratory manipulation and amenable to experimental evolution (Karpicka‐Ignatowska et al. 2021; Kuczyński et al. 2016; Laska et al. 2021; Skoracka, Rector, and Hein 2018; Skoracka et al. 2022). WCM mites are dispersed passively by wind currents. When exposed to wind, their response indicates either a propensity for dispersal or a lack of it (Kuczyński et al. 2020; Laska et al. 2019).

### 2.2 Experimental Evolution

We used lineages of WCM MT‐1 that differed in their host specialisation. To minimise background noise arising from genetic heterogeneity, we applied a replicated experimental evolution design to obtain lineages that were ecologically distinct yet comparable, as they originated from the baseline colony. These lineages were obtained through over 100 generations of experimental adaptation under contrasting host regimes.

The baseline colony was established in November 2017 using 26 WCM MT‐1 populations collected from wheat fields in nine geographically distant locations across Poland. To confirm their MT‐1 genotype, individuals from all of the field‐collected populations were barcoded using mtDNA COI. Approximately 1000 adult females from each population were then combined to form the baseline colony, which was maintained for 4 weeks under constant conditions (22°C–24°C, 12:12 D/N, 40% RH) before initiating the evolutionary experiment. This procedure ensured high genetic diversity while guaranteeing that all experimental lineages stemmed from a common gene pool.

To examine the effects of different host‐use regimes, we established two evolutionary treatments: (i) a specialist regime, in which populations were maintained exclusively on wheat ( *Triticum aestivum* ); and (ii) a generalist regime, in which populations alternated between wheat and barley ( *Hordeum vulgare* ). Six independent lineages (replicates) were established for each regime. Each lineage began with 300 WCM MT‐1 individuals, which were sampled from a baseline colony and transferred to clean potted wheat plants. Every 3 weeks—corresponding to approximately three generations at 27°C (Karpicka‐Ignatowska et al. 2021)—around 300 individuals from each lineage were transferred to fresh plants according to their assigned regime: wheat only for specialists, and an alternating wheat/barley for generalists. This meant that generalists spent 3 weeks either on wheat or barley. All lineages were maintained under controlled conditions (27°C, 16:8‐h light:dark cycle, 60% relative humidity) in separate isolators to prevent cross‐contamination.

One specialist lineage failed during the experiment. After concluding the evolutionary experiment, we assessed the fitness of five specialists and six generalists across a variety of plant species to verify their host specialisation. The generalists exhibited increased niche breadth, as evidenced by their high population growth rates (fitness) on multiple plant species, including those not encountered during experimental evolution. In contrast, the specialists showed strong adaptation to wheat and poor performance on alternative hosts. All lineages exhibited consistent patterns of host adaptation within their respective evolutionary regimes (Skoracka et al. 2022).

### 2.3 Experimental Design

We created the experimental environments using four plant species: wheat (*Triticum aestivum*, W), barley ( *Hordeum vulgare* , B), smooth brome ( *Bromus inermis* , S—previously demonstrated to be a less suitable host, Skoracka et al. 2022) and oats ( *Avena sativa* , O—previously demonstrated to be an unsuitable host, authors—unpublished).

Familiarity was defined as whether a lineage had encountered a given plant species during experimental evolution. Wheat was familiar to both specialists and generalists; barley, only to generalists; and smooth brome and oats, to neither.

Our key experimental variables were:

- Host specialisation: specialists and generalists produced through experimental evolution, as described above.
- Current habitat context: the plant fragment on which mites were placed, classified as either familiar or unfamiliar.
- Target habitat information: olfactory cues (kairomones) delivered to the mites, originating from familiar, unfamiliar or the mixtures of plants. This variable comprises:
  - Signal complexity: the number of kairomones provided (ranging from a single cue to mixtures of two or three),
  - Signal‐to‐noise ratio: the log‐odds of familiar to unfamiliar cues.

We only used kairomones emitted by the plants themselves, without any mechanical interference or induction by feeding mites. This ensured that the cues reflected plant identity alone, without confounding effects of herbivory, competition or population density.

The response variable was dispersal rate, measured as the proportion of individuals departing from the experimental arena after exposure to clean wind (control) or wind containing kairomones. The area representing the current environment consisted of a 1 cm^2^ plant fragment placed on agar blocks prepared from a modified in vitro culture medium (Karpicka‐Ignatowska et al. 2019; Murashige and Skoog 1962). At least 10 WCM females were acclimatised for 30 min before testing. The arena was then placed in an olfactometer connected to an Olympus SZX7 stereomicroscope equipped with an Olympus SC50 camera.

The olfactometer comprised tightly connected elements that ensured the controlled delivery of kairomones to the arena: an air pump acting as the kairomone generator, and a chamber containing a plant that served as the kairomone source. The system also included chambers filled with activated charcoal to provide clean air input or output (Figures S1 and S2 in Supporting Information S1). Mites were exposed to a to 3.7 m/s wind containing either plant volatiles or clean wind for 3 min. After exposure, the number of individuals remaining in the experimental arena was recorded.

We conducted two complementary experiments.

In Experiment 1, we investigated how familiarity with a single target kairomone modulates dispersal, given the current host. Departure rates were recorded for specialists and generalists when they were placed on W, B or S and were exposed to single kairomones produced by W, B, O or S or clean air (control).

In Experiment 2, we tested how the mixtures of kairomones affect dispersal. This was achieved by recording the departure rates of specialists and generalists as they settled on W and were exposed to mixtures containing two or three kairomones: W‐S, S‐O, B‐S‐O or W‐S‐O. The mixtures contained either only unfamiliar cues or a combination of familiar and unfamiliar cues.

A uniform replication structure was applied across experiments. For each trial, at least 10 females were transferred from a given lineage to the arena. We used five specialist and six generalist lineages. Each of the 19 experimental variants (combinations of current and target environments) was replicated five times per lineage, giving 25 replicates per variant for specialists (five lineages × five repetitions) and 30 replicates per variant for generalists (six lineages × five repetitions). In total, the study comprised 1045 experimental trials. A detailed breakdown of these variants is provided in Table S1 (Supporting Information S1).

### 2.4 Statistical Analysis

We fitted Generalised Linear Mixed Models (GLMMs) using the ‘glmmTMB’ package in R (Brooks et al. 2017). We modelled the proportion of dispersers using a beta‐binomial distribution to account for overdispersion.

To disentangle the effects of information familiarity from specific chemical identities (W, B, S, O), we fitted two complementary models to the Experiment 1 data.

- *Model 1A: Contextual familiarity*
  This model tested whether dispersal is driven by the cue recognition relative to evolutionary experience, regardless of its specific identity. We recoded kairomone treatments into a three‐level factor: Control (no cue), Familiar (experienced during evolution) and Unfamiliar (novel). Fixed effects included this familiarity factor, the relational status of the current plant environment (Familiar vs. Unfamiliar), host specialisation (Generalist vs. Specialist) and their interactions.
- *Model 1B: Chemical identity*
  This model tested the response to specific semiochemicals to determine if particular plants elicit stronger dispersal responses. The fixed effects included the specific kairomone identity (five levels: Control, W, B, O, S), current host plant species, host specialisation and their interactions.

Both models included a random intercept for lineage identity to account for variation arising from independent evolutionary trajectories. Statistical inference relied on Type II Wald *χ* ^2^ tests, and post hoc comparisons were conducted using estimated marginal means (Searle et al. 1980).

To investigate how information complexity and reliability influence dispersal decisions, we analysed the response to kairomone mixtures (Experiment 2). We restricted this analysis to the familiar wheat environment (W) and excluded the clean air control to isolate the effects of signal modulation. We fitted two complementary models, both assuming a beta‐binomial distribution for the response.

- *Model 2A: Signal complexity*
  This model tested the effect of signal quantity by asking how the sheer volume of sensory information affects dispersal. We modelled dispersal rate as a function of the total number of received kairomones. We allowed for group‐specific intercepts and slopes by interacting the number of kairomones with a combined grouping factor derived from host specialisation (Generalist vs. Specialist) and kairomone familiarity (presence of any familiar cue vs. purely unfamiliar cues).
- *Model 2B: Signal reliability*
  This model tested the effect of information quality. We defined the signal‐to‐noise (SN) ratio as the log‐odds of encountering evolutionarily familiar versus an unfamiliar kairomones: $\mathrm{SN}=\log_{2}\left(\left(F+1\right)/\left(U+1\right)\right)$, where F is the number of familiar kairomones and U is the number of unfamiliar kairomones (N−F) within the mixture. This transformation creates a symmetric scale where 0 represents a neutral information state (1:1 ratio), positive values indicate a dominance of familiar signals and negative values indicate a dominance of noise (unfamiliar cues). The addition of 1 prevents infinite values when cues of one type are absent.

We modelled dispersal rate as a function of the SN ratio, allowing for distinct intercepts and slopes for generalists and specialists to evaluate differences in sensitivity to signal degradation. We assessed whether dispersal rate changed with signal attributes by estimating the linear trends (slopes on the link scale) for each group. To determine if sensitivity to signal complexity or reliability differed between groups (e.g., Generalist vs. Specialist), we performed pairwise comparisons of these slopes using estimated marginal trends (‘emtrends’ function in the ‘emmeans’ package).

The code and data used in the analysis are available on GitHub (https://github.com/popecol/disinfo) and Zenodo (https://doi.org/10.5281/zenodo.16910509).

## 3 Results

### 3.1 Context‐Dependent Dispersal Responses

**FIGURE 1:**
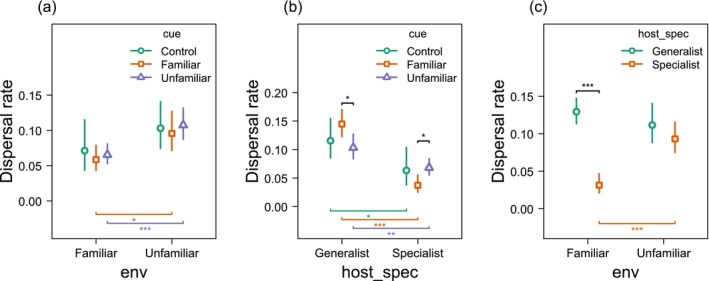
Interactive effects of host specialisation, olfactory cues, and current environment on dispersal rates. Estimated marginal means (±95% CI) of dispersal probability derived from a generalised linear mixed model assuming a beta‐binomial distribution. (a) Dispersal rates in relation to the current environment (Familiar vs. Unfamiliar) and kairomone cues. Kairomone treatments were defined as: Control (no kairomones), Familiar (cues encountered during experimental evolution), and Unfamiliar (novel cues never encountered before). (b) Dispersal rates in relation to host specialisation (Generalist vs. Specialist) and kairomone cues. (c) Dispersal rates in relation to the current environment and host specialisation. Significance notation: Top brackets indicate significant pairwise contrasts between grouping levels (legend categories) within a specific *x*‐axis treatment. Bottom brackets (coloured by group) indicate significant pairwise contrasts between *x*‐axis levels within a specific grouping category. Significance levels: *p < 0.05; **p < 0.01; ***p < 0.001.

Dispersal was not governed by a generalised response to olfactory information (main effect of cue: *χ* ^2^ = 2.6, d.f. = 2, *p* = 0.27). Instead, dispersal probability was highly context‐dependent, shaped by the interaction between the current host plant and evolutionary history (Table 1; Figure 1, Figure S3).

**TABLE 1:** Summary of Type II Wald *χ* ^2^ tests examining the effects of olfactory cue familiarity, current environment context, and host specialisation on mite dispersal rate. The data were analysed using a Generalised Linear Mixed Model (GLMM) with a beta‐binomial distribution to account for overdispersion. ‘Olfactory cue context’ describes the relationship between the mite's evolutionary history and the cue (Control, Familiar, or Unfamiliar). ‘Current environment’ describes the relationship between the mite's history and the plant substrate (Familiar or Unfamiliar). Significant *p*‐values (< 0.05) are indicated in bold.

Effect	$\chi^{2}$	d.f.	*p*
Olfactory cue context (Control, Familiar, Unfamiliar)	2.59	2	0.2735
Current environment (Familiar or Unfamiliar)	11.60	1	**0.0007**
Specialisation (Generalist or Specialist)	34.32	1	**< 0.0001**
Olfactory cue: Current environment	2.74	2	0.2540
Olfactory cue: Specialisation	14.70	2	**0.0006**
Current environment: Specialisation	19.55	1	**< 0.0001**
Olfactory cue: Current environment: Specialisation	5.06	2	0.0796

In contrast to the inconsistent effects of olfactory cues, current host plant familiarity exerted a strong and consistent influence (*χ* ^2^ = 11.6, d.f. = 1, *p* < 0.001). Overall, mites were less likely to disperse from evolutionarily familiar hosts (OR = 0.61, 95% CI: 0.46–0.81, *z* = −3.5, *p* < 0.001; Figure 1a). However, a significant host × specialisation interaction (*χ* ^2^ = 19.5, d.f. = 1, *p* < 0.001) revealed that this response was driven by specialists. Generalists dispersed at similar rates from familiar and unfamiliar plants (OR = 1.18, 95% CI: 0.88–1.59, *z* = 1.1, *p* = 0.27), whereas specialists dispersed significantly more from unfamiliar hosts (OR = 0.32, 95% CI: 0.20–0.51, *z* = −4.8, *p* < 0.0001). Consequently, on familiar hosts, generalists maintained higher baseline dispersal than specialists (OR = 4.58, 95% CI: 2.97–7.07, *z* = 6.9, *p* < 0.0001; Figure 1c). Together, these results indicate that specialists exhibited a much stronger residency response to host familiarity than generalists.

Responsiveness to olfactory information was contingent on host specialisation (Cue × Specialisation: *χ* ^2^ = 14.7, d.f. = 2, *p* < 0.001). Generalists exhibited higher overall dispersal rates than specialists (OR = 2.37, 95% CI: 1.79–3.13, *z* = 6.1, *p* < 0.001) and displayed divergent reaction norms (Figure 1b). Specifically, generalists were more likely to disperse from familiar cues relative to unfamiliar ones (OR = 1.47, 95% CI: 1.03–2.10, *z* = 2.54, *p* = 0.030). Conversely, specialists dispersed more frequently when exposed to unfamiliar cues (OR = 0.53, 95% CI: 0.31–0.92, *z* = −2.71, *p* = 0.019). This opposing directionality accounts for the non‐significant main effect of kairomones: the meaning of the same cue category differed fundamentally between generalists and specialists.

Analysis of specific chemical identities (Model 1B) reinforced these patterns. Specialists did not respond to any cue relative to the clean‐air control (*p* > 0.1), maintaining uniformly low dispersal. In contrast, generalists responded more strongly to wheat and barley (familiar hosts) than to the control (*p* = 0.029 and *p* = 0.013, respectively), whereas cues from oats and smooth brome elicited no significant response (Table S2, Figure S4). Finally, the non‐significant three‐way interaction (Cue × Environment × Specialisation) suggests that responses to the current environment and olfactory cues operated additively.

### 3.2 Dispersal in Response to the Signal Complexity and Reliability

**FIGURE 2:**
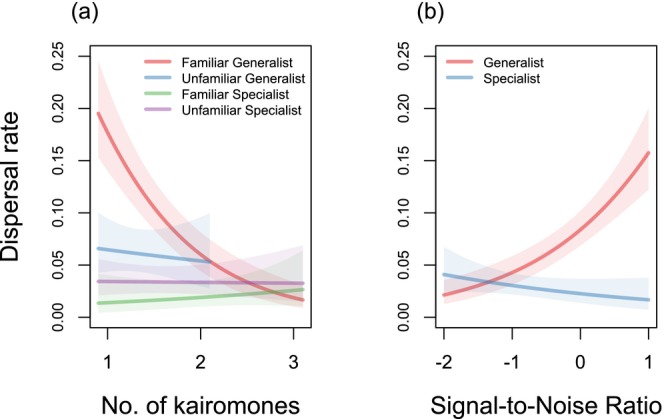
The dispersal rate as a function of (a) the number of kairomones received simultaneously and (b) the signal‐to‐noise ratio (SN). In panel (a), lines are fitted for groups representing combinations of cue type (Familiar or Unfamiliar) and host specialisation degree (Generalist or Specialist). Cues are regarded as ‘Unfamiliar’ if none of the kairomones in the delivered mixture have been previously encountered; ‘Familiar’ refers to those instances where at least one of the kairomones has been encountered previously. In panel (b), the relationship is shown for the two levels of host specialisation, where the SN ratio represents the log‐odds of the number of familiar to unfamiliar kairomones within the delivered mixture. Negative values of this metric indicate a dominance of noise (unfamiliar cues), and positive values indicate a dominance of familiar signals. Zero represents a state of maximum confusion (1:1 ratio). The shaded regions in both panels represent the 95% confidence intervals for the fitted lines.

Olfactory complexity influenced dispersal, but only for generalists exposed to mixtures containing familiar cues (Table S3). In this group, dispersal propensity declined sharply with increasing kairomone richness (Slope: *β* = −1.21, SE = 0.17, *p* < 0.001). Conversely, generalists exposed to unfamiliar mixtures and specialists in all contexts were unresponsive to signal complexity (*p* > 0.4 for all slopes; Figure 2a).

Information reliability (signal‐to‐noise ratio; SN) also elicited lineage‐specific responses (Table S4). Generalist dispersal increased with the SN ratio (*β* = 0.72, SE = 0.20, *p* < 0.0001), whereas specialists showed no significant response (*p* = 0.12). Consequently, generalists were significantly more sensitive to information reliability than specialists (contrast of slopes: 1.02, SE = 0.23, *z* = 4.46, *p* < 0.0001) (Figure 2b).

## 4 Discussion

Our experiments revealed three key patterns: (i) host familiarity drives strong philopatry in specialists but not in generalists; (ii) cue familiarity has the opposite effect on these two evolutionary strategies; and (iii) information load and reliability only influence dispersal in generalists. Although dispersal in passive dispersers ultimately depends on the departure phase, it may still involve some behavioural control and be initiated in response to environmental signals. For passively dispersing arthropods, kairomones can act as informative cues that guide take‐off decisions. These volatiles can signal deteriorating conditions in the current habitat, favourable abiotic conditions for transfer, or the presence of more attractive environments nearby (Bilton et al. 2001; Bonte et al. 2007; Birkett et al. 2004; Bruce et al. 2005; Magalhães et al. 2022, 2018).

Our experiments showed that the passive dispersal in phytophagous eriophyoid mites was not governed by a universal reaction to olfactory information, as evidenced by the insignificant main effect of kairomone cues. Instead, take‐off decisions were strongly contextual, emerging from a complex interplay between the current habitat and the organism's evolutionary history. A consistent residency effect occurred in both lineages: mites were markedly less likely to disperse when situated on an evolutionarily familiar host plant. However, the strength of this effect depended critically on the niche breadth. Specialists displayed a pronounced reluctance to leave familiar hosts and only dispersed readily when placed on unfamiliar plants, with little further modulation by cue identity (Figure 1c, Figure S4). In contrast, generalists maintained a high baseline dispersal rate, adjusting their dispersal behaviour according to the olfactory context: single familiar kairomones increased take‐off in generalists, whereas mixtures enriched with unfamiliar cues suppressed it (Figures 1b and 2a). Together, these patterns indicate that generalists use olfactory context to fine‐tune risk while specialists adopt a ‘stay‐unless‐necessary’ strategy. Understanding these divergent strategies is essential for predicting how landscape heterogeneity shapes specialisation, dispersal and consequently the invasive potential of pest populations (see Box 1).

BOX 1 **Practical implications for sensory‐based pest management.**

Many passively dispersing herbivorous arthropods, such as the wheat curl mite (*Aceria tosichella*), are major agricultural pests. Understanding the mechanisms underlying their dispersal, particularly those influencing their propensity to depart, is critical not only from an ecological and evolutionary perspective but also for developing innovative crop protection strategies. Such strategies could include engineering crops for endogenous resistance or treating them with semiochemicals to reduce their appeal to pests (Bruce et al. 2005). Specifically, identifying which chemical cues discourage or encourage pest dispersal can facilitate the development of effective and sustainable control and management strategies. For instance, using natural scents derived from non‐host plants could disrupt the ‘informed’ component of take‐off, thereby restricting pest spread into high‐value crops.

Our findings suggest that pest management can be improved by accounting for the interaction between a pest's niche breadth and the sensory landscape of the field. A key conclusion is that specialists and generalists respond differently to cues from their current and target environments. The experimental conditions under which our lineages evolved—homogeneous (monocultures) versus fluctuating (crop rotation) host regimes—correspond to real‐world agricultural practices. Specialists, which evolved under stable conditions, exhibited low dispersal rates and a strong ‘residency’ response to familiar hosts. In nature, such monocultures may produce ‘resident’ pests that are less likely to disperse but may utilize local resources more efficiently, making them more destructive within a single field. Conversely, generalists that evolved under environmental heterogeneity (rotation) exhibited high departure rates, making them more effective at infesting neighbouring fields. It transpires that within a single species, populations can react differently to the level of temporary environmental heterogeneity, which translates into their potential for dispersal and, consequently, their invasive potential.

Our results demonstrate that the high dispersal propensity of generalists is inhibited by complex mixtures of unfamiliar cues. Thus, intercropping or the application of non‐host volatiles could ‘mask’ the target environment by lowering the signal‐to‐noise ratio. By manipulating this ratio (e.g., by increasing crop diversity or leaving strips of natural vegetation between crops), practitioners could effectively trap generalists on their current host or delay their take‐off, reducing the rate of landscape‐wide infestation.

Generally, our findings suggest that agricultural practices act as an unintended selection pressure on pests and their propensity to spread. Specialist and generalist lineages of the same species likely commingle in agroecosystems, where ‘resident’ specialists cause severe localised damage and ‘migratory’ generalists drive rapid expansion. Therefore, monitoring the degree of host specialisation within a local population is vital. For specialist‐dominated populations, strategies should focus on endogenous resistance and localised control. For generalist‐dominated populations, management must be landscape‐wide, utilising sensory‐based Integrated Pest Management (IPM). Using natural scents as ‘olfactory barriers’ can exploit the generalist's sensitivity to future habitat cues to suppress their spread. Ultimately, understanding how agricultural practices influence the integration of ‘push’ (current host quality) and ‘pull’ (target host information) allows us to predict and mitigate the spread of invasive species by designing ‘noisy’ sensory landscapes that suppress informed departure.

### 4.1 Information From the Current and Target Environments

Theory predicts that passive dispersers should remain on current familiar hosts since aerial dispersal carries a high risk of ending up in an unsuitable habitat (Bonte et al. 2012; Teller et al. 2015). Our results partly support this expectation. The wheat‐adapted specialists indeed ‘played it safe’: they largely remained on wheat, where their performance was consistently high and only dispersed readily when placed on unfamiliar hosts such as barley or smooth brome, where their fitness declined (Skoracka et al. 2022). This behaviour aligns with the classic principle that organisms should not leave a high‐quality habitat when the expected benefits of doing so are low (MacArthur 1972).

By contrast, generalists showed no such home‐site loyalty. Their baseline departure rate remained high, regardless of whether they were placed on familiar or unfamiliar plants. This does not imply that generalists failed to discriminate between cue types. Rather, it reflects the fact that their broader ecological niche reduces the potential costs of dispersal (Dicke 2000). Generalists are more likely to land on a suitable host plant than specialists are, and they perform well on non‐preferred plants, such as smooth brome (Skoracka et al. 2022). Hence, for generalists, spreading offspring across multiple potential habitats is a viable bet‐hedging strategy that does not rely heavily on information from the current host.

In addition to evaluating the conditions of their current environment, organisms may benefit from identifying signals that provide information about potential future habitats (Nichols et al. 2020). Here, specialists and generalists responded very differently to target cues. Wheat‐adapted specialists behaved like risk‐averse, locally adapted phenotypes: they primarily left when the current plant was unfamiliar (Figure 1c), rather than in response to target cues (Figure 1b). Although these specialists dispersed slightly more when exposed to unfamiliar kairomones, our detailed chemical‐identity analysis showed that their overall departure rates from all plant species remained uniformly low and statistically indistinguishable from those in the clean‐air control (Figure S4b). Therefore, olfactory discrimination did not influence the take‐off decisions of specialists under the conditions tested.

Specialist departure thresholds are likely shaped not only by plant‐derived odours but also by density‐dependent pressures such as kin competition or resource depletion that we did not manipulate here. These drivers are well known to promote dispersal in many taxa (Poethke and Hovestadt 2002; Bonte et al. 2012; Bitume et al. 2013) and could increase the relevance of olfactory information for specialists under more stressful conditions. Future experiments integrating density manipulations with multi‐cue olfactory contexts may therefore clarify when specialists rely on chemical information and when they adopt a strictly philopatric strategy (cf. Bruce et al. 2005; Bruce and Pickett 2011).

In contrast, generalists displayed the opposite tendency. While their baseline dispersal remained high across cue types, the plant species‐level analysis revealed clear selectivity: volatiles from wheat and barley (the plants on which they evolved) facilitated take‐off more than the control, whereas cues from oats or smooth brome did not (Figure S4b). This pattern mirrors those observed in other passive generalists, such as the spider mite, *Tetranychus urticae*, which moves preferentially toward volatiles of its own host plant (Gotoh et al. 1993), and Lepidoptera larvae that use plant odours to guide their settlement after windborne dispersal (Zalucki et al. 2002).

### 4.2 Environmental Heterogeneity

Natural foragers rarely encounter isolated host plumes; instead, they must extract meaningful information from a chemically complex background consisting of volatiles from multiple plant species (Beyaert and Hilker 2014; Bruce et al. 2005). In line with field surveys indicating that high chemical diversity can dilute herbivore attacks (Salazar et al. 2016), our mixture experiment revealed a clear ‘noise’ effect, but only for the generalists. Wheat specialists maintained a consistently low departure rate regardless of the number of contributing plant species cues or the signal‐to‐noise ratio. This pattern suggests that specialists either ignore the olfactory background or rely almost exclusively on cues from their current host when deciding whether to take off.

Generalists, however, responded strongly to the olfactory composition. Their take‐off was the highest when a single familiar cue was presented alone, but dispersal declined as mixture became increasingly enriched with unfamiliar cues. This suggests that greater signal heterogeneity increases uncertainty and reduces the incentive to leave. One plausible mechanism is ratio‐specific recognition, whereby generalists track the proportions of volatiles associated with familiar hosts. Introducing non‐host odours can distort these ratios (or mask key components), eroding the emigration trigger (Bruce et al. 2005; Bruce and Pickett 2011). In chemically complex environments, this signal‐to‐noise penalty would therefore favour rapid departure upon detecting a clean, familiar scent, but suppress further take‐off once that scent becomes embedded within a heterogeneous blend (Beyaert and Hilker 2014; Salazar et al. 2016). This decision‐making process is consistent with the broader host range of *A. tosichella* generalists, who maintain fitness on non‐preferred hosts (Skoracka et al. 2022), and with theoretical predictions that broad‐niche organisms can afford, and often benefit from, frequent movement in variable environments (Büchi and Vuilleumier 2014; Ravigné et al. 2024; Ward et al. 2001). Thus, in noisy landscapes, generalists appear to pay a signal‐to‐noise penalty that initiates rapid departure upon detecting a lone host scent, but they show reduced readiness to disperse once that scent is masked by heterospecific odours.

### 4.3 Degree of Host Specialisation and Response to Cues

In our experiments, the mites' degree of host specialisation governed their departure behaviour more than the mere presence of kairomones. This aligns with joint‐evolution models, in which niche breadth and dispersal form a feedback loop: specialisation is paired with philopatry, whereas a broad diet reduces the costs of ‘rolling the dice’ and therefore selects for high dispersal (Ravigné et al. 2024). Empirically, wheat‐adapted specialists left the plant only half as often as their generalist sister lineages (overall OR ≈ 0.42), even after accounting for cue type and current host, consistent with the predicted asymmetry. Whether this pattern generalises across phytophagous arthropods remains uncertain. Comparative and experimental surveys find that roughly 60% of cases show higher movement in generalists, yet exceptions exist: some specialists, typically those with well‐developed movement capacities and perceptual range, disperse more (Dapporto and Dennis 2013; Dennis et al. 2000; Nieminen 1996). Conversely several studies have reported no clear relationship (Peterson and Denno 1998; Van Zandt and Mopper 1998). For passively transported taxa, direct empirical tests remain particularly scarce. These inconsistencies in the literature likely originate from the difficulty of accounting for multiple environmental contexts simultaneously, an inherent limitation of broad comparative studies (e.g., Stevens et al. 2012, 2013). By experimentally isolating the effects of current habitat quality, target olfactory cues and evolutionary history, our approach demonstrates that dispersal is not a fixed trait of a lineage, but rather a flexible response to integrated information. This underscores the need for a multi‐contextual framework that incorporate both laboratory and natural field studies (Box 2) in order to uncover the mechanisms underlying informed dispersal.

BOX 2 **Sensory ecology: From lab signals to olfactory noise in nature.**

Our findings demonstrate that dispersal in *Aceria tosichella*—a significant agricultural pest—is not a random escape but an informed decision emerging from the integration of multiple information sources. While our laboratory setup provided stable kairomone streams to isolate the mechanistic underpinnings of take‐off behaviour, natural agricultural landscapes present turbulent, highly dynamic odour plumes in which the signal‐to‐noise ratio fluctuates rapidly (Beyaert and Hilker 2014). For microscopic passive dispersers, the effective detection range of these cues is likely restricted to very short spatial scales, specifically within the laminar boundary layer immediately above the leaf surface (Dicke 2000). Consequently, plant‐derived kairomones in the field are unlikely to act as long‐distance attractants. Instead, we suggest they function as local modulators of departure decisions, triggering take‐off when mites encounter upwind cues during questing behaviour. This parallels the strategy of other passive dispersers, such as ballooning spiders, which interpret local abiotic and biotic signals before initiating their aerial transport (Bonte et al. 2012; Cho et al. 2018).

In natural settings, mites rarely encounter isolated olfactory signals. Instead, they experience a chemically crowded environment shaped by plant community composition, stress responses, microbial interactions, and atmospheric conditions (Bruce et al. 2005). Our results highlight that specialists and generalists extract information from these chemically complex environments in fundamentally different ways. In monocultures, specialists encounter high‐signal, low‐noise environments where host volatiles are reliable indicators of habitat quality, thereby reinforcing philopatry and residency. In contrast, the inhibitory effect of complex kairomone blends on generalist dispersal (Experiment 2) suggests an adaptive ‘wait‐and‐see’ strategy in heterogeneous landscapes. By remaining philopatric when olfactory information is ambiguous or ‘noisy’, generalists may avoid the high mortality risk associated with dispersing into a diverse but potentially unsuitable landscape (Bruce and Pickett 2011).

This sensory‐contextual framework helps reconcile why previous broad‐scale surveys have often found inconsistent links between niche breadth and dispersal propensity (e.g., Alzate et al. 2017; Stevens et al. 2013). Many previous analyses focused on morphological or life‐history traits without explicitly considering how organisms perceive and integrate environmental information. Our results point instead to the interaction between evolutionary history and the sensory milieu as the missing dimension in dispersal theory. Ultimately, while a lineage's niche breadth dictates its baseline propensity for informed dispersal, the local olfactory context determines whether passive take‐off is initiated. By integrating sensory ecology with evolutionary history, we move from viewing dispersal as a fixed life‐history trait toward understanding it as a dynamic, adaptive response to the informational complexity of real‐world landscapes.

The theory also predicts that specialists should rely more heavily on host‐derived volatiles, given that the cost of settling in an unsuitable habitat is high. Yet, electrophysiological studies often reveal similar receptor breadth in both specialists and generalists (Bruce et al. 2005). Our results echo this paradox: specialists did not respond more strongly to familiar cues, whereas generalists fine‐tuned their already high dispersal according to olfactory context. Together, these findings imply that diet breadth established the baseline propensity to departure, while chemical information modulates this tendency in a manner that depends on, rather than determines, specialisation. To obtain a fuller picture, future work should track both take‐off and settlement in passive dispersers and assess whether differences in niche breadth are reflected in the underlying olfactory architecture or merely in the decision‐making processes that organisms use to integrate environmental information.

## Author Contributions

Kamila Zalewska conceptualised and designed the study with Lechosław Kuczyński, Anna Skoracka, Ewa Puchalska, Mariusz Lewandowski assistance. Kamila Zalewska ran experiments and collected data. Lechosław Kuczyński performed statistical analyses. Lechosław Kuczyński, Anna Skoracka, Dries Bonte, Ewa Puchalska, Mariusz Lewandowski and Kamila Zalewska critically interpreted the results. Anna Skoracka, Lechosław Kuczyński and Dries Bonte wrote the manuscript with assistance of Ewa Puchalska, Mariusz Lewandowski, Kamila Zalewska. All authors contributed substantially to revisions, read and approved the final version of the manuscript.

## Funding

This work was supported by Narodowe Centrum Nauki, 2019/35/N/NZ8/03377.

## Conflicts of Interest

The authors declare no conflicts of interest.

## Supporting information


**Table S1:** Experimental variants made to test the dispersal rate of the wheat curl mite (WCM) in the response to cues from the current and target environments consisting of plant species: B = barley, O = oats, S = smooth brome, W = wheat and 0 = no cues. Each variant was independently repeated five times for each of the five specialist lineages and each of the six generalist lineages. This resulted in a total of 25 replicates per variant for specialists (5 lineages × 5 repetitions) and 30 replicates per variant for generalists (6 lineages × 5 repetitions), totalling 1045 experimental trials (including 10 females per trial).
**Figure S1:** Schematic diagram of the olfactometer. (A, E) chamber filled with activated charcoal; (B) chamber with a plant being the source of kairomones; (C) chamber in which tested wheat curl mite (WCM) individuals were placed; (D) plant fragment with mite individuals placed on agar blocks: experimental arena—current environment.
**Figure S2:** The equipment for testing dispersal responses to kairomones (olfactometer).
**Supporting Information: S2** Estimated marginal means contrasts.
**Supporting Information: S3** Supplementary Results.
**Figure S3:** Main effects of olfactory cues, current environment and host specialisation on dispersal rates. Estimated marginal means (±95% CI) of dispersal probability derived from a generalised linear mixed model assuming a beta‐binomial distribution. (a) Dispersal rates across kairomone treatments: Control (no kairomones), Familiar (cues encountered during experimental evolution) and Unfamiliar (novel cues). (b) Dispersal rates in Familiar versus Unfamiliar current environments. (c) Dispersal rates for Generalist versus Specialist host strategies. Significance notation: Brackets indicate significant pairwise contrasts between factor levels (*p < 0.001).
**Table S2:** Summary of Type II Wald *χ* ^2^ tests examining the effects of specific kairomone identity, current host plant species and host specialisation on mite dispersal propensity. Data were restricted to single‐source kairomone treatments (Experiment 1). The model assumes a beta‐binomial distribution for the response. ‘Kairomone identity’ includes five levels: Control (clean air), wheat (W), barley (B), oats (O) and smooth brome (S). ‘Current host plant’ includes three levels: W, B and S. Significant *p*‐values (< 0.05) are indicated in bold.
**Figure S4:** Interactive effects of host specialisation, olfactory cues and current environment on dispersal decisions. Estimated marginal means (±95% CI) of dispersal probability derived from a generalised linear mixed model (GLMM) assuming a beta‐binomial distribution. (a) Dispersal rates in response to the current environment (*x*‐axis) and kairomone cues (legend). (b) Dispersal rates in relation to host specialisation and kairomone cues. (c) Dispersal rates in relation to host specialisation and the current environment. Abbreviations: Cues (kairomones): control (none); W (wheat); B (barley); S (smooth brome); O (oats). Significance notation: Top brackets indicate significant pairwise contrasts between legend categories within a specific *x*‐axis level. Bottom brackets (coloured by group) indicate significant contrasts between x‐axis levels within a specific legend category. Significance levels: *p < 0.05; **p < 0.01; **p < 0.001.
**Table S3:** Estimated regression parameters from a Generalised Linear Mixed Model (GLMM) relating signal complexity (number of kairomones) to mite dispersal propensity. The analysis was restricted to the familiar wheat environment. The model fitted separate intercepts and slopes for each combination of host specialisation and kairomone familiarity. ‘Familiar kairomones’ indicates mixtures containing known cues (i.e., wheat and barley for Generalists; wheat for Specialists); ‘Unfamiliar kairomones’ indicates mixtures of novel cues (i.e., oats, smooth brome for Generalists; oats, smooth brome, barley for Specialists). Significant *p*‐values (< 0.05) are indicated in bold.
**Table S4:** Estimated regression parameters from a Generalised Linear Mixed Model (GLMM) relating signal reliability: Signal‐to‐Noise (SN) ratio to mite dispersal rate. The model fitted separate intercepts and slopes for Generalists and Specialists. The SN ratio is defined as the log‐odds of the number of familiar to the number of unfamiliar kairomones within the mixture. Significant *p*‐values (< 0.05) are indicated in bold.

## Data Availability

The data that support the findings and the code used to produce the results of this study are openly available on GitHub (https://github.com/popecol/disinfo) and Zenodo (https://doi.org/10.5281/zenodo.16910509).
