# Supplementary material for: Niche Breadth and Olfactory Context Shape Informed Passive Dispersal
Source: Ecol Lett. 2026 Mar 31;29(4):e70373. doi: 10.1111/ele.70373 (PMC13039768; doi:10.1111/ele.70373)
Supplement: Supplementary file 1 — Table S1: Experimental variants made to test the dispersal rate of the wheat curl mite (WCM) in the response to cues from the current and target environments consisting of plant species: B = barley, O = oats, S = smooth brome, W = wheat and 0 = no cues. Each variant was independently repeated five times for each of the five specialist lineages and each of the six generalist lineages. This resulted in a total of 25 replicates per variant for specialists (5 lineages × 5 repetitions) and 30 replicates per variant for generalists (6 lineages × 5 repetitions), totalling 1045 experimental trials (including 10 females per trial). Figure S1: Schematic diagram of the olfactometer. (A, E) chamber filled with activated charcoal; (B) chamber with a plant being the source of kairomones; (C) chamber in which tested wheat curl mite (WCM) individuals were placed; (D) plant fragment with mite individuals placed on agar blocks: experimental arena—current environment. Figure S2: The equipment for testing dispersal responses to kairomones (olfactometer). Supporting Information: S2 Estimated marginal means contrasts. Supporting Information: S3 Supplementary Results. Figure S3: Main effects of olfactory cues, current environment and host specialisation on dispersal rates. Estimated marginal means (±95% CI) of dispersal probability derived from a generalised linear mixed model assuming a beta‐binomial distribution. (a) Dispersal rates across kairomone treatments: Control (no kairomones), Familiar (cues encountered during experimental evolution) and Unfamiliar (novel cues). (b) Dispersal rates in Familiar versus Unfamiliar current environments. (c) Dispersal rates for Generalist versus Specialist host strategies. Significance notation: Brackets indicate significant pairwise contrasts between factor levels (*p < 0.001). Table S2: Summary of Type II Wald χ 2 tests examining the effects of specific kairomone identity, current host plant species and host specialisation on mite dispersal propen [file ELE-29-0-s001.pdf]

Supporting Information

Niche breadth and olfactory context shape informed passive dispersal

Kamila Zalewska[1,2], Anna Skoracka[1*], Dries Bonte[3], Ewa Puchalska[4], Mariusz Lewandowski[4], Lechosław Kuczyński[1]

[1]Population Ecology Lab, Institute of Environmental Biology, Faculty of Biology, Adam Mickiewicz University, Uniwersytetu Poznańskiego 6, 61-614 Poznań, Poland.

[2]Center for Advanced Technology, Adam Mickiewicz University, Uniwersytetu Poznańskiego 10, 61-614 Poznań

[3]Department of Biology, Ghent University, K.L. Ledeganckstraat 35 9000 Gent, Belgium

[4] Warsaw University of Life Sciences, Department of Plant Protection, Warsaw, Poland

*corresponding author: Anna Skoracka, Uniwersytetu Poznańskiego 6, 61-614 Poznań, Poland, phone: 0048783309590, e-mail: skoracka@amu.edu.pl

Supporting Information 1 – Supplementary Methods

Table S1. Experimental variants made to test the dispersal rate of the wheat curl mite (WCM) in the response to cues from the current and target environments consisting of plant species: B=barley, O=oats S=smooth brome, W=wheat, and 0=no cues. Each variant was independently repeated five times for each of the five specialist lineages and each of the six generalist lineages. This resulted in a total of 25 replicates per variant for specialists (5 lineages x 5 repetitions) and 30 replicates per variant for generalists (6 lineages x 5 repetitions), totalling 1,045 experimental trials (including 10 females per trial).

Experiment	Current environment	Target environment	Remarks
1	W	0	Control
1	S	0	Control
1	B	0	Control
1 & 2	W	W	1 kairomone
1 & 2	W	B	1 kairomone
1 & 2	W	S	1 kairomone
1 & 2	W	O	1 kairomone
1	B	W	1 kairomone
1	B	B	1 kairomone
1	B	S	1 kairomone
1	B	O	1 kairomone
1	S	W	1 kairomone
1	S	B	1 kairomone
1	S	S	1 kairomone
1	S	O	1 kairomone
2	W	W-S	2 kairomones
2	W	S-O	2 kairomones
2	W	B-S-O	3 kairomones
2	W	W-S-O	3 kairomones

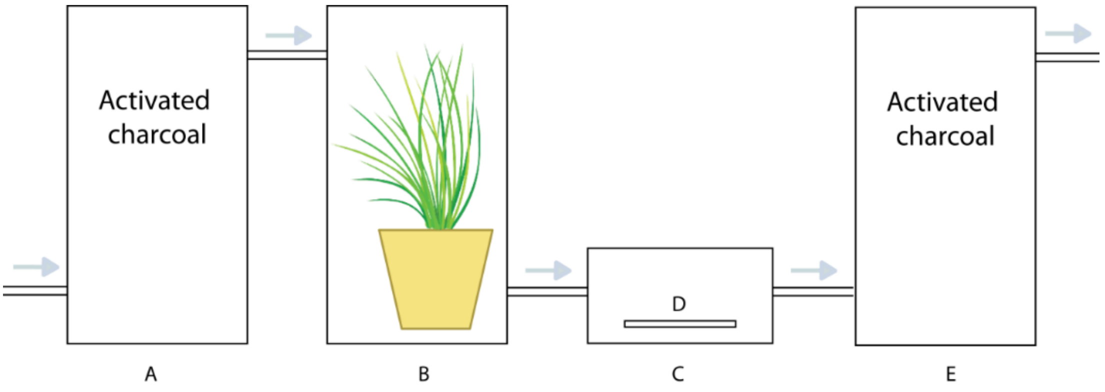

Fig. S1. Schematic diagram of the olfactometer. A, E – chamber filled with activated charcoal; B – chamber with a plant being the source of kairomones; C – chamber in which tested wheat curl mite (WCM) individuals were placed; D – plant fragment with mite individuals placed on agar blocks: experimental arena – current environment.

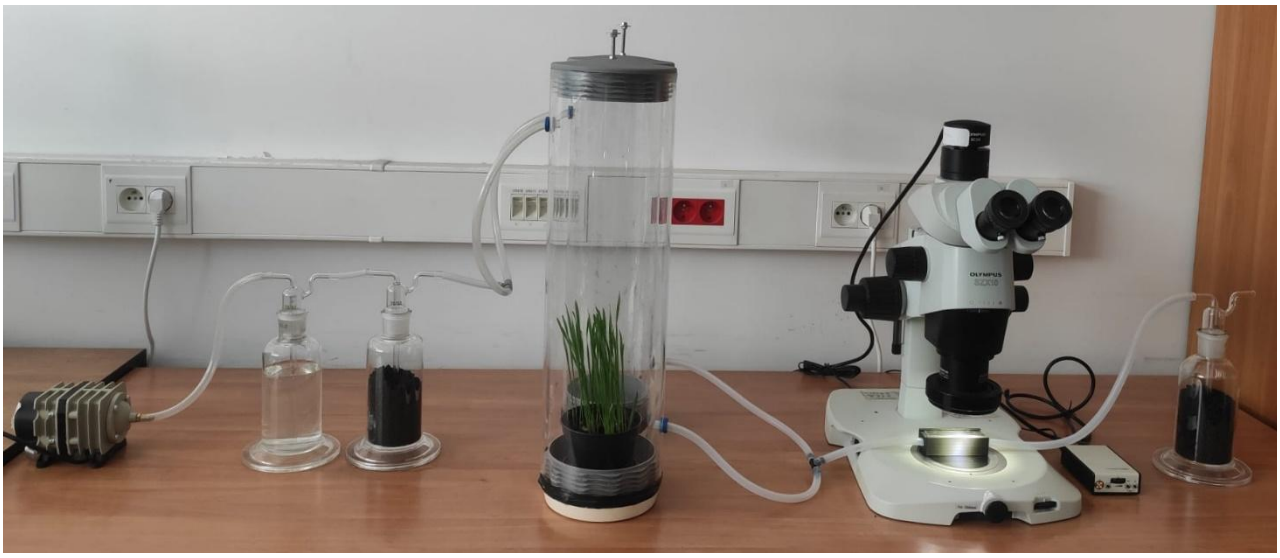

Fig. S2. The equipment for testing dispersal responses to kairomones (olfactometer).

Supporting Information 2 – Estimated marginal means contrasts

1. Marginal effects

1.1. Kairomones (cues)

```
contrast               odds.ratio    SE  df null z.ratio p.value
 control / familiar         1.159 0.228 Inf    1   0.748  0.7347
 control / unfamiliar       1.023 0.184 Inf    1   0.127  0.9911
 familiar / unfamiliar      0.883 0.123 Inf    1  -0.892  0.6451

Results are averaged over the levels of: env, host_spec
P value adjustment: tukey method for comparing a family of 3 estimates
Tests are performed on the log odds ratio scale
```

1.2. Current environment

```
contrast               odds.ratio     SE  df null z.ratio p.value
 familiar / unfamiliar      0.612 0.0869 Inf    1  -3.462  0.0005

Results are averaged over the levels of: cue, host_spec
Tests are performed on the log odds ratio scale
```

1.3. Specialisation

```
contrast odds.ratio    SE  df null z.ratio p.value
 G / S         2.37 0.337 Inf    1   6.056  <.0001

Results are averaged over the levels of: cue, env
Tests are performed on the log odds ratio scale
```

2. Conditional effects: 2-way interactions

2.1. Kairomones : Current environment

```
cue = control:
 contrast              odds.ratio     SE  df null z.ratio p.value
 familiar / unfamiliar      0.669 0.2150 Inf    1  -1.251  0.2110

cue = familiar:
 contrast              odds.ratio     SE  df null z.ratio p.value
 familiar / unfamiliar      0.588 0.1340 Inf    1  -2.338  0.0194

cue = unfamiliar:
 contrast              odds.ratio     SE  df null z.ratio p.value
 familiar / unfamiliar      0.582 0.0944 Inf    1  -3.339  0.0008

Results are averaged over the levels of: host_spec
Tests are performed on the log odds ratio scale
```

2.2. Kairomones : Specialisation

```
cue = control:
 contrast odds.ratio    SE  df null z.ratio p.value
 G / S          1.93 0.622 Inf    1   2.044  0.0409

cue = familiar:
 contrast odds.ratio    SE  df null z.ratio p.value
 G / S          4.36 0.993 Inf    1   6.473  <.0001

cue = unfamiliar:
 contrast odds.ratio    SE  df null z.ratio p.value
 G / S          1.57 0.255 Inf    1   2.794  0.0052

Results are averaged over the levels of: env
Tests are performed on the log odds ratio scale
```

2.3. Current environment : Specialisation

```
host_spec = G:
 contrast              odds.ratio     SE  df null z.ratio p.value
 familiar / unfamiliar      1.183 0.1800 Inf    1   1.108  0.2677

host_spec = S:
 contrast              odds.ratio     SE  df null z.ratio p.value
 familiar / unfamiliar      0.316 0.0759 Inf    1  -4.798  <.0001

Results are averaged over the levels of: cue
Tests are performed on the log odds ratio scale
```

3. Conditional effects: 3-way interactions

```
cue = control, host_spec = G:
 contrast              odds.ratio     SE  df null z.ratio p.value
 familiar / unfamiliar      2.170 0.7390 Inf    1   2.276  0.0228

cue = familiar, host_spec = G:
 contrast              odds.ratio     SE  df null z.ratio p.value
 familiar / unfamiliar      0.797 0.1540 Inf    1  -1.172  0.2413

cue = unfamiliar, host_spec = G:
 contrast              odds.ratio     SE  df null z.ratio p.value
 familiar / unfamiliar      0.958 0.2240 Inf    1  -0.184  0.8539

cue = control, host_spec = S:
 contrast              odds.ratio     SE  df null z.ratio p.value
 familiar / unfamiliar      0.206 0.1130 Inf    1  -2.890  0.0039

cue = familiar, host_spec = S:
 contrast              odds.ratio     SE  df null z.ratio p.value
 familiar / unfamiliar      0.434 0.1780 Inf    1  -2.032  0.0421

cue = unfamiliar, host_spec = S:
 contrast              odds.ratio     SE  df null z.ratio p.value
 familiar / unfamiliar      0.353 0.0794 Inf    1  -4.632  <.0001

Tests are performed on the log odds ratio scale
```

Supporting Information 3 – Supplementary Results

1. The main effect of kairomones

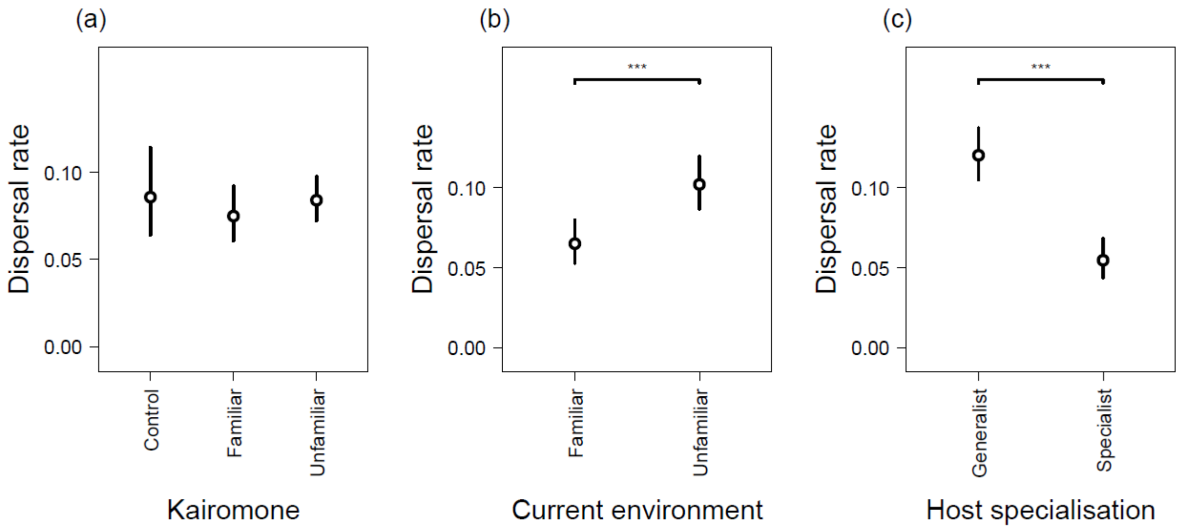

Fig. S3. Main effects of olfactory cues, current environment, and host specialisation on dispersal rates. Estimated marginal means (± 95% CI) of dispersal probability derived from a generalized linear mixed model assuming a beta-binomial distribution. (a) Dispersal rates across kairomone treatments: Control (no kairomones), Familiar (cues encountered during experimental evolution), and Unfamiliar (novel cues). (b) Dispersal rates in Familiar versus Unfamiliar current environments. (c) Dispersal rates for Generalist versus Specialist host strategies. Significance notation: Brackets indicate significant pairwise contrasts between factor levels (∗ P<0.05; ∗∗ P<0.01; ∗∗∗ P<0.001).

2. Analysis of chemical identity (Model 2B)

This analysis corroborated the findings regarding cue familiarity (Table S2) while refining our understanding of how host specialisation mediates responses to specific olfactory cues (Fig. S4b). Generalists exhibited significantly elevated dispersal rates in the presence of cues from familiar hosts compared to the clean air control (wheat: p=0.029, barley: p=0.013). Furthermore, their response to familiar cues was significantly stronger than their response to cues from smooth brome (p<0.001 for both contrasts), indicating a specific recognition of familiar host volatiles. Meanwhile, generalist responses to cues from unfamiliar plants (smooth brome and oats) were indistinguishable from the control (p=0.17 and p=0.99, respectively). In contrast, specialists displayed generally low dispersal propensity, with no significant differences detected between any specific kairomone treatment and the control (p>0.1).

Table S2. Summary of Type II Wald $\chi 2$ tests examining the effects of specific kairomone identity, current host plant species, and host specialisation on mite dispersal propensity. Data were restricted to single-source kairomone treatments (Experiment 1). The model assumes a beta-binomial distribution for the response. "Kairomone identity" includes five levels: Control (clean air), wheat (W), barley (B), oats (O), and smooth brome (S). "Current host plant" includes three levels: W, B, and S. Significant p-values (<0.05) are indicated in bold.

Effect	χ^2	d.f.	p-value
Kairomone identity (Control, W, B, O, S)	16.7	4	**0.0022**
Current host plant (W, B, S)	61.9	2	**<0.0001**
Specialisation (Generalists, Specialists)	31.4	1	**<0.0001**
Kairomone identity : Current host plant	10.2	8	0.2561
Kairomone identity : Specialisation	30.2	4	**<0.0001**
Current host plant: Specialisation	8.6	2	**0.0137**
Kairomone identity : Current host plant: Specialisation	10.2	8	0.2499

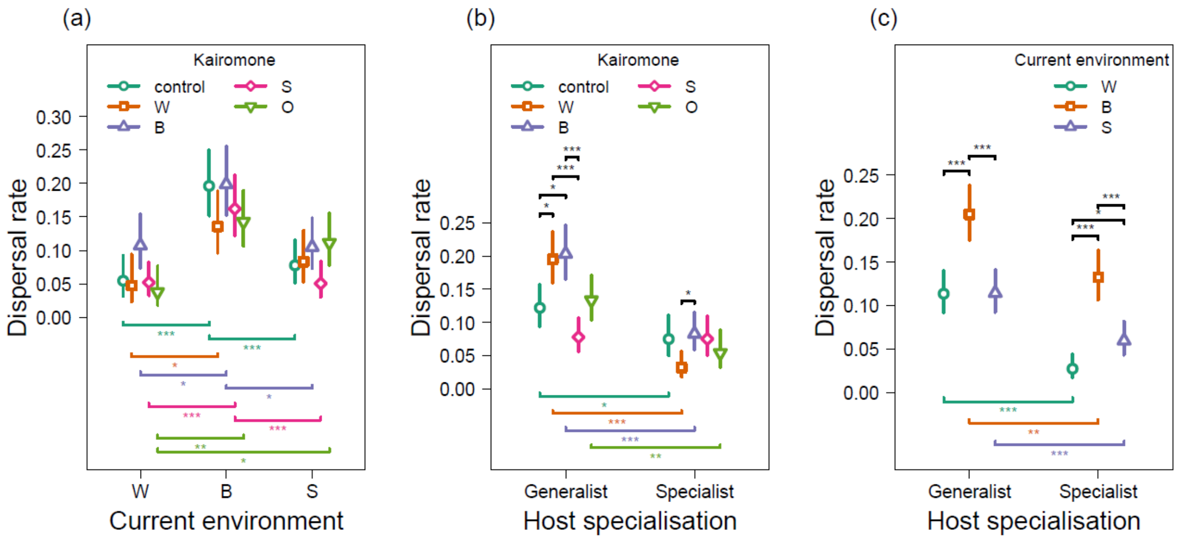

Fig. S4. Interactive effects of host specialisation, olfactory cues, and current environment on dispersal decisions. Estimated marginal means (± 95% CI) of dispersal probability derived from a generalized linear mixed model (GLMM) assuming a beta-binomial distribution. (a) Dispersal rates in response to the current environment (x-axis) and kairomone cues (legend). (b) Dispersal rates in relation to host specialisation and kairomone cues. (c) Dispersal rates in relation to host specialisation and the current environment. Abbreviations: Cues (kairomones): control (none); W (wheat); B (barley); S (smooth brome); O (oats). Significance notation: Top brackets indicate significant pairwise contrasts between legend categories within a specific x-axis level. Bottom brackets (coloured by group) indicate significant contrasts between x-axis levels within a specific legend category. Significance levels: $*$ P<0.05; $**$ P<0.01; $***$ P<0.001.

3. Dispersal in response to the signal complexity and reliability

Table S3. Estimated regression parameters from a Generalized Linear Mixed Model (GLMM) relating signal complexity (number of kairomones) to mite dispersal propensity. The analysis was restricted to the familiar wheat environment. The model fitted separate intercepts and slopes for each combination of host specialisation and kairomone familiarity. "Familiar kairomones" indicates mixtures containing known cues (i.e., wheat and barley for Generalists; wheat for Specialists); "Unfamiliar kairomones" indicates mixtures of novel cues (i.e., oats, smooth brome for Generalists; oats, smooth brome, barley for Specialists). Significant p-values (<0.05) are indicated in bold.

	**Estimate**	**SE**	**z-value**	**p-value**
**Intercepts**				
Generalist-Familiar kairomones	-0.33	0.27	-1.21	0.2250
Generalist-Unfamiliar kairomones	-2.48	0.52	-4.80	**<0.0001**
Specialist-Familiar kairomones	-4.56	0.90	-5.09	**<0.0001**
Specialist-Unfamiliar kairomones	-3.31	0.42	-7.82	**<0.0001**
**Slopes (response to the number of kairomones)**				
Generalist-Familiar kairomones	-1.21	0.17	-6.92	**<0.0001**
Generalist-Unfamiliar kairomones	-0.19	0.37	-0.52	0.6050
Specialist-Familiar kairomones	0.31	0.38	0.80	0.4210
Specialist-Unfamiliar kairomones	-0.02	0.23	-0.11	0.9160

Table S4. Estimated regression parameters from a Generalized Linear Mixed Model (GLMM) relating signal reliability: Signal-to-Noise (SN) ratio to mite dispersal rate. The model fitted separate intercepts and slopes for Generalists and Specialists. The SN ratio is defined as the log-odds of the number of familiar to the number of unfamiliar kairomones within the mixture. Significant p-values (<0.05) are indicated in bold.

	**Estimate**	**SE**	**z-value**	**p-value**
**Intercepts**				
Generalist	-2.39	0.11	-21.9	**< 0.0001**
Specialist	-3.77	0.26	-14.6	**< 0.0001**
**Slopes (response to SN)**				
Generalist	0.72	0.11	6.24	**< 0.0001**
Specialist	-0.31	0.20	-1.55	0.1220
